# Combination chemotherapy with carboplatin, capecitabine and epirubicin (ECarboX) as second- or third-line treatment in patients with relapsed ovarian cancer: a phase I/II trial

**DOI:** 10.1038/sj.bjc.6602879

**Published:** 2005-11-22

**Authors:** C Rothermundt, R Hubner, T Ahmad, I Gibbens, C Keyzor, T Habeshaw, S Kaye, M Gore

**Affiliations:** 1 The Royal Marsden Hospital NHS Foundation Trust, Fulham Road, London SW3 6JJ, UK; 2 The Institute of Cancer Research, 123 Old Brompton Road, London SW7 3RP, UK; 3 The Royal Marsden Hospital NHS Foundation Trust, Downs Road, Sutton, Surrey SM2 5PT, UK; 4 Roche Products Limited, 40 Broadwater Road, Welwyn Garden City, Herts AL7 3AY, UK

**Keywords:** capecitabine, carboplatin, epirubicin, ovarian cancer, relapse

## Abstract

Platinum-based combination chemotherapy has been proven to be superior to single-agent platinum in the treatment of relapsed ovarian cancer after a treatment-free interval of more than 6 months. A response rate of 41% was previously reported by our group using a combination of epirubicin, cisplatin and 5-FU in patients who relapsed within 12 months, we therefore assessed a similar, but more convenient combination of epirubicin, carboplatin and capecitabine in this phase-I/II trial. In total, 18 patients with recurrent epithelial ovarian carcinoma, who had not received more than two lines of chemotherapy and the treatment-free interval exceeded 6 months were treated with carboplatin AUC5, epirubicin 50 mg m^−2^ and capecitabine at several dose levels on continuous 21 day cycles and 14 of 21 day cycles. Patients were assessed for toxicity and by CT and CA-125 for response. The overall response rate was 61.1%, with three complete and eight partial responses. Grade 3/4 haematological toxicity was seen in 10 out of 18 patients and caused dose reductions and treatment delays. The combination of epirubicin, carboplatin and capecitabine showed good activity but caused excessive toxicity. A phase-II trial using carboplatin and capecitabine is underway.

Ovarian cancer remains the number one cause of death from gynaecological neoplasms in the Western world (Greenlee *et al*, 2001). Response rates to first-line chemotherapy in women with ovarian cancer are high but most patients relapse and need further treatment. Recurrent disease is incurable (Ozols, 2002); however, many patients can obtain good palliation from further treatment, particularly if they obtain an objective response (Cannistra, 2002).

Platinum-based combination chemotherapy has been proven to be superior to single-agent carboplatin in patients with so-called chemo-sensitive relapsed ovarian cancer in terms of response rate and progression-free survival in two randomised studies and overall survival in one of them (Parmar *et al*, 2003; Pfisterer *et al*, 2005). Chemo-sensitive relapse is usually defined as one that occurs after a platinum-free interval of over 6 months.

However, there is uncertainty concerning which agent to add to carboplatin in this situation: data on the activity of single-agent non-platinum therapy have shown similar efficacy for paclitaxel (Piccart *et al*, 2000), docetaxel (Kaye *et al*, 1997), epirubicin (Havsteen *et al*, 1996), gemcitabine (Shapiro *et al*, 1996), liposomal doxorubicin (Muggia *et al*, 1997; Gordon *et al*, 2001), etoposide (Rose *et al*, 1998), topotecan (Gore *et al*, 2002), oxaliplatin (Chollet *et al*, 1996), and vinorelbine (Sorensen *et al*, 2001). There is one analysis that shows superiority for caelyx over topotecan in patients with a progression-free interval >6 months but this was a subset analysis (Gordon *et al*, 2004).

Our group has shown that patients re-challenged with platinum using a combination of epirubicin, cisplatin and continuous infusional 5-fluoruracil (ECF) for recurrent ovarian cancer have a response rate of 41%. In this study, the patients had relapsed within 12 months of platinum treatment (Ahmed *et al*, 1995). Considerable morbidity can be associated with an indwelling central venous catheter for the administration of 5-FU, such as pneumothorax at the time of insertion, sepsis or thrombosis. We therefore decided to investigate the substitution of continuous intravenous 5-FU in the ECF regimen with capecitabine (Xeloda), an orally bio-available fluoropyrimidine carbamate. The conversion of the prodrug capecitabine to 5-fluoruracil is dependent on thymidine phosphorylase, an enzyme preferentially expressed in malignant cells, and is considered tumour-selective (Ishikawa *et al*, 1998; Schilsky, 2000). Capecitabine has been demonstrated to have activity in relapsed ovarian cancer in three recently published phase II trials (Boehmer and Jaeger, 2002; Vasey *et al*, 2003; Rischin *et al*, 2004). In addition, we replaced the cisplatin with carboplatin to make the regimen more convenient for patients.

The aim of this study was to identify the maximum tolerated dose (MTD) of capecitabine in combination with epirubicin and carboplatin in patients with relapsed ovarian cancer. We planned to identify safe doses of the drugs in this combination so that a phase-III trial could be performed. We also evaluated the response rate to carboplatin, capecitabine and epirubicin (ECarboX) as a secondary end point.

## PATIENTS AND METHODS

Patients were eligible if they had recurrent, histologically verified epithelial ovarian carcinomas, had not received more than two lines of chemotherapy and the treatment-free interval between the cessation of the previous chemotherapy and documented relapse exceeded 6 months.

Additional inclusion criteria were: age 18–80 years, ECOG performance status 0–2, life expectancy >3 months, measurable disease as assessed by CT in accordance with WHO guidelines, GFR⩾60 ml min^−1^ as measured by EDTA clearance or 24 h urine collection, bilirubin <2 times ULN, adequate bone marrow function with WBC>3000 *μ*l^−1^, neutrophils >1500 *μ*l^−1^, platelets >100 000 *μ*l^−1^. Fully informed written consent was obtained from all patients.

Patients were excluded for any serious uncontrolled medical condition, a significantly abnormal ECG or cardiac history of having a LVEF<LLN measured by MUGA scan or echocardiogram, or any medical or psychiatric condition, which would impair the patients’ ability to give informed consent.

The protocol and the consent form were approved by the local institutional ethics committee and protocol review board.

### Treatment plan

Carboplatin was administered at a dose level of AUC5 intravenously over a 1 h infusion once every 3 weeks, epirubicin was given as an intravenous bolus at a dose of 50 mg m^−2^ once every 3 weeks. Administration of capecitabine was by the oral route and the doses to be used in successive cohorts of patients were: capecitabine 375 mg m^−2^ BD (level 1), 500 mg m^−2^ BD (level 2) daily throughout a 21-day cycle, and 625 mg m^−2^ BD (level 3) for days 1–14 of a 21-day cycle. The decision to increase from level 1 to 2 was based on the toxicity experienced in the first cycle of each cohort of three patients. Escalation was to be carried out if dose-limiting toxicity was not seen in the first three patients in each cohort. There were two additional dose levels (−1 and −2). Patients treated at these dose levels received capecitabine for days 1–14 on a 21-day cycle at 500 mg m^−2^ BD (level −1) and 375 mg m^−2^ BD (level −2) daily, respectively. These dose levels were introduced when it became clear that a 14 rather than 21-day schedule for capecitabine was necessary if this three-drug regimen was to be administered safely. All patients received prophylactic antiemetic therapy prior to administration of carboplatin.

Six cycles of chemotherapy were planned for each responding patient or those with stable disease. Criteria for withdrawal from the study were as follows: intolerable side effects as judged by the investigator or the patient, patient decision to discontinue treatment for any reason, recurrent grade 3 or 4 drug-related toxicity despite dose modifications, progression of tumour and serious allergic reactions to any of the study drugs. Dose reductions for haematological toxicity were performed according to the protocol. Patients who withdrew from the study were assessed for toxicity and response. Dose limiting toxicity (DLT) was defined as grade IV myelotoxicity, which was either prolonged (>7 days) or complicated by bleeding or infection, or grade III nonhaematological toxicity occurring in the first cycle.

### Assessment of safety and efficacy

Toxicity and adverse events were reported according to NCIC-CTC after each cycle.

Response was assessed by CT according to WHO criteria after cycle 3 and 6; in addition, tumour marker CA-125 was used to monitor response and was measured before each cycle. Complete response was defined as normalisation of radiological appearances and normalisation of CA-125.

## RESULTS

In total, 18 patients with relapsed epithelial ovarian carcinoma and a median age of 58 years (range, 43–70 years) were enrolled from October 2002 until June 2004. A total of 13 patients had only received one line of chemotherapy previously. The other five patients received ECarboX as third-line treatment (Table 1). All patients had a platinum-free interval >6 months and were thus considered platinum-sensitive. The median platinum-free interval was 18.6 months with a range of 8.7–55.4 months. One of the patients had previously received radiotherapy to the brain for cerebral metastases.

Three patients were assigned to dose level 1 (capecitabine 375 mg m^−2^ BD q21) and five patients to dose level 2 (capecitabine 500 mg m^−2^ BD q21). Following a protocol amendment, one patient was treated at dose level 3 (capecitabine 625 mg m^−2^ BD q14). Six patients received dose level −1 (capecitabine 500 mg m^−2^ BD q14) and three patients dose level −2 (capecitabine 375 mg m^−2^ BD q14), respectively.

### Toxicity

At dose level 1, no DLT was experienced in the first cycle, although one patient had grade 3 neutropenia and required subsequent dose delays and dose reductions as per protocol. Another patient experienced grade 3 diarrhoea and recurrent episodes of neutropenia on cycles 2 and 4. She required a >50% dose reduction of capecitabine and epirubicin and was treated off-study after cycle 4 due to the delays of treatment. She did however have a complete response.

At dose level 2, the cohort was expanded to five patients after one patient had experienced a DLT (bleeding) during the first cycle and required a 1-week delay of cycle 2. Two out of five patients suffered from prolonged neutropenia following the first cycle, which prevented retreatment with carboplatin on day 22. One of these patients had another 3-week delay of her third cycle due to grade 3 neutropenia and therefore came off-trial; she had progressive disease after cycle three. Another patient experienced grade 3 fatigue.

Following a protocol amendment due to the high rate of prolonged grade 3 neutropenia, we treated patients with a shortened schedule of capecitabine (14 out of 21 days). One patient was treated at 625 mg m^−2^ BD for 14 days on a 21-day cycle (level 3). She developed small bowel obstruction following the administration of the first cycle of chemotherapy and required surgery; she became neutropenic while awaiting surgery. The patient was subsequently treated off-study with another four courses at reduced dose and achieved a complete response. No further patients were enrolled at this dose level as this severe adverse event was felt to represent DLT.

Three out of six patients on dose level −1 required delays of day 22 due to prolonged grade 3 neutropenia, one had grade 3 thrombocytopenia and one grade 3 palmar-plantar-syndrome (PPS). All other side effects were infrequent. After another dose de-escalation, three patients were treated at level −2: one patient had grade 4 stomatitis and one grade 3 neutropenia.

A total of 37 cycles of chemotherapy (out of 77 delivered on study) had to be postponed due to toxicity. The median delay was 2 weeks per patient (range 0–5 weeks).

The MTD was dose level 2 with capecitabine given at 500 mg m^−2^ BD for 21 days of a 21-day cycle. This schedule was not considered suitable for taking forward to a larger multicentre trial because of the frequent incidence of prolonged neutropenia. 14-day courses of capecitabine were better tolerated; this schedule, however, still caused Grade 3/4 haematological toxicity requiring dose reductions and delays in 60% (six out of 10) of the patients (Table 2).

### Efficacy

In total, 11 patients (61.1%) showed either a complete (three patients; 16.7%) or a partial response (eight patients; 44.4%), five (27.7%) had stable disease. Only two (11.1%) of the patients had progressive disease on treatment.

The median progression-free survival was 8.3 months, the median follow-up time was 18 months (range 7.3–27.2 months). Median overall survival has not yet been reached and 14 patients are still alive.

## DISCUSSION

We have performed a phase I/II trial using carboplatin, epirubicin and capecitabine in platinum-sensitive patients with relapsed epithelial ovarian cancer. The rationale behind this regimen was that we had previously demonstrated a high response rate in patients treated with ECF for relapsed ovarian cancer at the Royal Marsden Hospital (Webb *et al*, 2000), and wanted to investigate the feasibility of a more convenient route of administration for a fluoropyrimidine. We used capecitabine instead of 5-FU as this enables delivery of the fluoropyrimidine in an oral form rather than via a continuous infusion requiring a PICC line, without compromising on efficacy as shown for colorectal cancer in randomised trials (Hoff *et al*, 2001; Van Cutsem *et al*, 2001). We initially chose to administer continuous capecitabine to mirror continuously infused 5-FU, but this proved impractical.

In addition, carboplatin has been shown to be equally active as cisplatin in advanced ovarian cancer (Swenerton *et al*, 1992; Taylor *et al*, 1994; Ozols *et al*, 2003), but is less toxic and easier to administer. This was the reason why we changed the cisplatin to carboplatin for the ECarboX regimen.

We found the ECarboX regimen to be active with three patients having a CR and eight patients a PR, giving an overall response rate (ORR) of 61.1%. CR was seen in patients at dose level 1 and dose level 3, PR was seen on all other dose levels. The median progression-free survival of 8.3 months is consistent with the results seen in this patient group in other phase I/II trials (Kose *et al*, 2005).

However, ECarboX proved problematic to deliver as prescribed. The overall toxicity was high in terms of haematological side effects, although surprisingly no major infectious complications arose from the neutropenic episodes. Prolonged myelotoxicity was a significant feature even when administration of capecitabine was shortened. All other side effects were infrequent and manageable.

One patient's treatment was complicated by a cerebrovascular event and a deep venous thrombosis during cycle two. She was taken off study and treated with single-agent carboplatin. There are reports suggesting capecitabine can induce acute coronary syndromes (Schnetzler *et al*, 2001; Frickhofen *et al*, 2002), but no reports of cerebrovascular events caused by capecitabine exist. It remains unclear whether this patient's CVA was related to chemotherapy. The patient who developed intestinal obstruction following the first course of treatment was thought to have disease progression rather than a side effect from the regimen.

The management of patients with platinum-sensitive relapsed ovarian cancer is receiving increasing attention. A retrospective analysis (Dizon *et al*, 2002) of patients who received carboplatin/paclitaxel at relapse demonstrated an OR of 69.7% and a progression-free interval of 13 months. A total of 61.9% of these patients had already received carboplatin/paclitaxel as first-line treatment. However, 77% had a treatment-free interval >12 months and therefore would be considered good-risk patients. Toxicity was acceptable although six patients required admission to hospital for adverse events.

The ICON4/AGO-OVAR-2.2 trial investigated carboplatin/paclitaxel *vs* conventional platinum-based chemotherapy in women with relapsed ovarian cancer (Parmar *et al*, 2003). In this trial, about 40% of patients had received a platinum/paclitaxel combination previously as first-line treatment. Response rates were higher in the carboplatin/paclitaxel arm (OR 66 *vs* 54%, *P*=0.06) as was the median progression-free survival (12 *vs* 9 months) and overall survival (29 *vs* 24 months, HR=0.82, *P*=0.02).

However, there may be problems in re-treating patients with carboplatin/paclitaxel at relapse when they have received this combination at first line particularly if their treatment-free interval is 6–12 months. These patients frequently have a degree of residual peripheral neuropathy and have only just regained a normal length of hair; repeating and possibly exacerbating the former toxicity in a short space of time is not acceptable to many patients and their physicians, especially since these patients have a rather poor prognosis. Carboplatin/gemcitabine is a treatment option for patients with a potentially platinum-sensitive relapse. In a randomised study conducted by the AGO in 356 patients, the median progression-free survival was 8.6 months for patients treated with carboplatin/gemcitabine and 5.8 months for those receiving single-agent carboplatin, the response rate in the combination arm was 47.2 and 30.9% for carboplatin alone (Pfisterer *et al*, 2005).

In the context of these different regimens, other combinations such as ECarboX are worthy of consideration. However, toxicity remains a major concern and the question that has to be raised is whether the potential risks outweigh the benefits.

One option would be to omit the anthracycline from the ECarboX regimen. Such an approach is supported by data from the Arbeitsgemeinschaft Gynaekologische Onkologie (AGO) group trial and the NSGO-EORTC-NCIC CTG gynaecological cancer intergroup trial. These were two large randomised trials of first-line chemotherapy, both of which have shown that the addition of epirubicin to carboplatin/paclitaxel is of no benefit (Kristensen *et al*, 2003; Du Bois *et al*, 2004).

The addition of epirubicin to a two-drug combination in the second-line setting may therefore be of doubtful value. Consequently we are currently evaluating the use of carboplatin in combination with capecitabine in a phase-2 trial. This two-drug combination may prove to be a suitable candidate for further randomised trials in relapsed ovarian cancer.

## Figures and Tables

**Table 1:** Patient characteristics

Total patients	18
Second line treatment	13
Third line treatment	5
Median age	58 years (range, 43–70 years)
Platinum-free interval	18.6 months (range, 8.7–55.4 months)

**Table 2:** Toxicities (Grade 3/4)

**Dose level**	**1**	**2**	**3**	**−1**	**−2**	**Summary**
Capecitabine (mg m^−2^ BD)	375 q21	500 q21	625 q14	500 q14	375 q14	
Patient number	*n*=3	*n*=5	*n*=1	*n*=6	*n*=3	*n*=18
Neutropenia grade	2	2	1	3	1	9
Thrombocytopenia grade				1		1
Stomatitis grade					1	1
Diarrhoea	1					1
Fatigue		1				1
PPS				1		1
Median delay (weeks) (range)	3 (0–4)	2 (1–4)	2	1 (0–2)	3 (1–5)	2 (0–5)
